# Segmentability Differences Between Child-Directed and Adult-Directed Speech: A Systematic Test With an Ecologically Valid Corpus

**DOI:** 10.1162/opmi_a_00022

**Published:** 2019-02-01

**Authors:** Alejandrina Cristia, Emmanuel Dupoux, Nan Bernstein Ratner, Melanie Soderstrom

**Affiliations:** Dept d’Etudes Cognitives, ENS, PSL University, EHESS, CNRS; 2 Dept d’Etudes Cognitives, ENS, PSL University, EHESS, CNRS; 3 INRIA; 4 FAIR Paris; Department of Hearing and Speech Sciences, University of Maryland; Department of Psychology, University of Manitoba

**Keywords:** computational modeling, learnability, infant word segmentation, statistical learning, lexicon

## Abstract

Previous computational modeling suggests it is much easier to segment words from child-directed speech (CDS) than adult-directed speech (ADS). However, this conclusion is based on data collected in the laboratory, with CDS from play sessions and ADS between a parent and an experimenter, which may not be representative of ecologically collected CDS and ADS. Fully naturalistic ADS and CDS collected with a nonintrusive recording device as the child went about her day were analyzed with a diverse set of algorithms. The difference between registers was small compared to differences between algorithms; it reduced when corpora were matched, and it even reversed under some conditions. These results highlight the interest of studying learnability using naturalistic corpora and diverse algorithmic definitions.

## INTRODUCTION

Although children are exposed to both child-directed speech (CDS) and adult-directed speech (ADS), children appear to extract more information from the former than the latter (e.g., Cristia, 2013; Shneidman & Goldin-Meadow, 2012). This has led some to propose that most or all linguistic phenomena are more easily learned from CDS than ADS (e.g., Fernald, 2000), with a flurry of empirical literature examining specific phenomena (see Guevara-Rukoz et al., 2018, for a recent review). Deciding whether the learnability of linguistic units is higher in CDS than ADS is difficult for at least two reasons: It is difficult to find appropriate CDS and ADS corpora; and one must have an idea of how children learn to check whether such a strategy is more successful in one register than the other. In this article, we studied a highly ecological corpus of CDS and child-overheard ADS with a variety of word segmentation strategies.

What is word segmentation? Since there are typically no silences between words in running speech, infants may need to carve out, or *segment*, word forms from the continuous stream. Several differences between CDS and ADS could affect word segmentation learnability. Caregivers may speak in a more variable pitch, leading both to increased arousal in the child (which should boost attention and overall performance; Thiessen, Hill, & Saffran, 2005) but also increased acoustic variability (which makes word identification harder; Guevara-Rukoz et al., 2018). To study word segmentation controlling for other differences (e.g., attention capture, fine-grained acoustics), we use computational models of word segmentation from phonologized transcripts. Word segmentation may still be easier in CDS than ADS:CDS is characterized by short utterances, including a high proportion of isolated words (e.g., Bernstein Ratner & Rooney, 2001, Soderstrom, 2007, pp. 508–509, and Swingley & Humphrey, 2018, for empirical arguments that frequency in isolation matters). Short utterances represent an easier segmentation problem than long ones, since utterance boundaries are also word boundaries, and proportionally more boundaries are provided for free. Other features of CDS may be beneficial or not depending on the segmentation strategy. For instance, CDS tends to have more partial repetitions than ADS (“Where’s the dog? There’s the dog!”), which may be more helpful to lexical algorithms (which discover recombinable units) than sublexical algorithms (that look for local breaks, such as illegal within-word phonotactics or dips in transition probability).

Previous modeling research documents much higher segmentation scores for CDS than ADS corpora (Batchelder, 1997, 2002; Daland & Pierrehumbert, 2011; Fourtassi, Borschinger, Johnson, & Dupoux, 2013). Most of this work compared CDS recorded in the home or in the lab (in the CHILDES database; MacWhinney, 2014), against lab-based corpora of adult–adult interviews including open-ended questions ranging from profession to politics (e.g., the Buckeye corpus; Pitt, Johnson, Hume, Kiesling, & Raymond, 2005). As a result, differences in segmentability could be due to confounded variables: Home recordings capture more informal speech than interviews do, with shorter utterances and reduced lexical diversity; moreover, since different researchers transcribed the CDS and ADS corpora, their criteria for utterance boundaries may not be the same.

Only two studies used matched corpora, which had been collected in the laboratory as mothers talked to their children and an experimenter. Batchelder (2002) applied a lexical algorithm onto the American English Bernstein Ratner corpus (Bernstein Ratner, 1984), and found a 15% advantage for CDS over ADS. Ludusan, Mazuka, Bernard, Cristia, and Dupoux (2017) applied two lexical and two sublexical algorithms to the Japanese-spoken Riken corpus (Mazuka, Igarashi, & Nishikawa, 2006), where the CDS advantage was between 2% and 10%. Still, it is unclear whether either corpus is representative of the CDS and ADS children hear every day. Being observed might affect parents’ CDS patterns, and thus segmentability. Moreover, ADS portions were elicited by unfamiliar experimenters, with whom mothers may have been more formal than in children’s typical overheard ADS. Experimenter-directed ADS can differ significantly from ADS addressed to family members even in laboratory settings, to the point that phonetic differences across registers are much reduced when using family-based (rather than experimenter-based) ADS as a benchmark (E. K. Johnson, Lahey, Ernestus, & Cutler, 2013). Since prior work used laboratory-recorded samples, it is possible that it has over- or misestimated differences in segmentability between CDS and ADS.

Therefore, we studied an ecological child-centered corpus containing both ADS and CDS. We followed Ludusan and colleagues (2017) by using both lexical and sublexical algorithms; in addition, we varied important parameters within these classes and further added two baselines. In all, we aimed to provide a more accurate and generalizable estimate of the size of segmentability differences in CDS versus ADS.

## METHODS

This article is reproducible thanks to the use of R, papaja, and knitr (Aust & Barth, 2015; R Core Team, 2015; Xie, 2015). Raw data, supplementary explanations on the methods, and supplementary analyses are also available (Cristia, 2018a).

### Corpus

The dataset consists of 104 recordings transcribed from the Winnipeg Corpus (Soderstrom, Grauer, Dufault, & McDivitt, 2018; Soderstrom & Wittebolle, 2013; some of the recordings are archived on homebank.talkbank.org—VanDam et al., 2016), gathered from 35 children (19 boys), aged between 13 and 38 months, recorded using the LENA system^1^ at home (14 children), at home daycare (6), or at daycare center (13), with one more child recorded both at home and home daycare. Soderstrom et al. (2018) report that, between 9 a.m. and 5 p.m., there were 1–4 adults in home recordings (median of 5-min units 1), 1–3 in home daycares (median 1), and 1–5 + in daycare centers (median 2). Although the caregivers’ sex was not systematically noted, a majority was female in all settings.

The first 15 min, one hr into the recording (min 60–75), were independently transcribed by two undergraduate assistants, who resolved any disagreements by discussion. Transcription was done at the lexical level adapting the CHILDES minCHAT guidelines for transcription (MacWhinney, 2009),^2^ without reproducing details of pronunciation (see Discussion). The transcribers also coded whether an utterance was directed to the target child, another child, an adult, or other, using content and context. Utterances directed to the target child constituted the CDS corpus; those directed to an adult constituted the ADS corpus.

Although LENA’s utterance boundaries were mostly accurate, coders sometimes split a single LENA segment into two utterances. Since LENA may miss boundaries, we always divided segments following human coding. Additionally, coders sometimes considered a sequence of segments as continuations of each other (6% of CDS utterances and 7% of ADS utterances).

We derived several versions of the ADS and CDS subcorpora crossing two factors (see Table 1 for characteristics). First, we used the automatic utterance boundaries provided by the LENA software (“A,” short for “automatic boundaries”), as well as combined together the text from segments labeled as continuations of each other by coders (“H” for “human boundaries”). Second, since performance is dependent on corpus size (see Bernard et al., 2018), we had three versions of each CDS corpus: the full one, a shortened CDS corpus to match the ADS corpus in number of words, and a shortened CDS corpus to match the ADS corpus in number of utterances. After crossing these two factors, performance could be compared between, on the one hand, ADS-A/H (ADS with automatic or human utterance boundaries), and, on the other hand, one of (1) CDS-A/H-full (corresponding full CDS corpus), (2) CDS-A/H-WM (cut at the same number of word tokens found in the corresponding ADS), or (3) CDS-A/H-UM (cut at the same number of utterances). As shown in the results, these different boundaries and matching conditions only clarify our main conclusions that CDS-ADS differences are very small.

**Table 1.** Characteristics of the ADS and CDS portions of the corpus, depending on whether the human or automatic utterance boundaries were considered.

	**Human**					**Automatic**				
	**Sylls**	**Tokens**	**Types**	**MTTR**	**Utts**	**Sylls**	**Tokens**	**Types**	**MTTR**	**Utts**
ADS	10,051	8,224	1,342	0.93	1,772	10,100	8,267	1,342	0.93	1,892
CDS	24,933	20,786	2,015	0.89	5,320	24,933	20,777	2,012	0.89	5,630

*Note*. Tokens differ for the Human versus Automatic because utterances where human coders (mistakenly) changed register within a continuation were dropped from the Human analyses. ADS = adult-directed speech; CDS = child-directed speech; Sylls = syllables; tokens and types refer to words; MTTR = Moving average Type to Token Ratio (over a sliding 10-word window); Utts = utterances.

### Processing and Evaluation

Scripts used for corpus preprocessing, phonologization, and segmentation are available (Cristia, 2018b). During preprocessing, all extraneous codes (such as marks for overlapping speech or lexical reference for unusual pronunciations) were removed, leaving only the orthographic representation of the adults’ speech. These were phonologized using the American English voice of Festival Text-to-Speech (Taylor, Black, & Caley, 1998), which provides phonemically based transcriptions, including syllable boundaries. These transcriptions emerge mostly from dictionary lookup, but the system can also perform grapheme–phoneme conversions for neologisms, which are frequent in child-directed speech. Spaces between words are removed from the resulting corpus to provide input to the algorithms. Each algorithm then returns the corpus with spaces where word boundaries are hypothesized.

Each algorithm (with default parameters, except as noted below) was run using the WordSeg package (Bernard et al., 2018), which also performs the evaluation. Due to space restrictions, we cannot provide fuller descriptions here, but we refer readers to Bernard et al. (2018), where the algorithms and the evaluation are explained. In a nutshell, both training and evaluation are done over the whole corpus because these algorithms are unsupervised, and thus there is no risk of overfitting. In the case of incremental algorithms, performance was calculated on an output corpus that represented the algorithm’s segmentation level in the last 20% of the data.

We provide pseudo-confidence intervals estimated as two standard deviations over 10 runs of resampling with replacement. That is, we created 10 versions of each corpus by resampling children’s transcripts to achieve approximately the same number of utterances as in the original. For example, in one of the runs, the ADS corpus may be composed of the data from child 2’s day 1, 24’s day 3, 5’s day 1, and so on. We then extracted the standard deviation in performance across resamples for each algorithm and corpus version.

For comparability with previous work, we focus on lexical token F-scores, derived by comparing the gold-standard version of the input against the parsed version returned by the algorithm. Precision measures what proportion of the word tokens posited by a given algorithm correspond to tokens found in the gold segmentation, while recall measures what proportion of the gold word tokens were correctly segmented by the algorithm. For instance, for the gold phrase “here we go,” if an algorithm returns “here wego,” precision is .5 (one out of two posited tokens is correct) and recall is .3 (one out of three gold words is correct). The overall F-score ranges from 0 to 1, as it is the harmonic mean of precision *P* and recall *R*, namely, 2 × (*P* × *R*/(*P* + *R*)), which is multiplied by 100 and reported as percentages here. Results for all other possible alternative metrics, and further discussion on these methods, are provided in the Supplemental Materials (Cristia, 2018a).

### Segmentation Algorithms

There were two variants for each of two popular sublexical algorithms. The first one, DiBS (short for Diphone-Based Segmentation; Daland & Pierrehumbert, 2011), posits word boundaries where phonotactic probabilities are low. The “gold” version (*phonotactic-gold*) sets the diphone probability threshold based on gold word boundaries. The unsupervised version (*phonotactic-unsupervised*) sets the threshold using utterance boundaries only. The phonotactics were computed on the concatenation of CDS and ADS versions of the corpus. The second algorithm, labeled TP, posits boundaries using transition probabilities between syllables, as proposed in Saffran, Aslin, and Newport (1996). The first version uses a relative dip in probabilities (henceforth *TP-relative*). That is, given the syllable sequence WXYZ, a boundary is posited between X and Y if the transition probability between the X-Y is lower than between W-X and Y-Z. The second version uses average transitions over all pairs of syllables in the corpus as the threshold (*TP-average*; Saksida, Langus, & Nespor, 2017).

Of the three lexical algorithms, two are variants on the Adaptor Grammar by M. Johnson and Goldwater (2009). In this system, there is a set of generic rules, such as “a word is a sequence of phonemes, an utterance is a sequence of words,” and the algorithm further learns, based on the corpus, particular instances of these rules (“d + o + g is a word”) as well as all of the rules’ probabilities. One variant relied on the simple rules just defined (*lexical-unigram*). The other variant, which we call *lexical-multigram*, is based on a more complicated rule set with hierarchically defined levels that are both smaller and larger than words (details in the Supplemental Materials, Cristia, 2018a; M. Johnson, Christophe, Dupoux, & Demuth, 2014). The third lexical algorithm, *lexical-incremental*, implements a very different approach (Monaghan & Christiansen, 2010). It processes the corpus one utterance at a time. For each, it checks whether the utterance contains a subsequence that is in its long-term lexicon; if so, it checks whether extracting that subsequence would result in phonotactically legal remainders (with phonotactics derived from the lexicon). Otherwise, the whole utterance is stored in its lexicon.

To these seven algorithms we add two baselines, introduced to provide segmentation results for relatively uninformed strategies. One posits word boundaries at utterance edges (henceforth *base-utt*). The other posits word boundaries at syllable edges (henceforth *base-syll*). The latter is likely to be effective for English CDS, which has a very high proportion of monosyllabic words (e.g., Swingley, 2005).

## RESULTS

**Figure 1.**
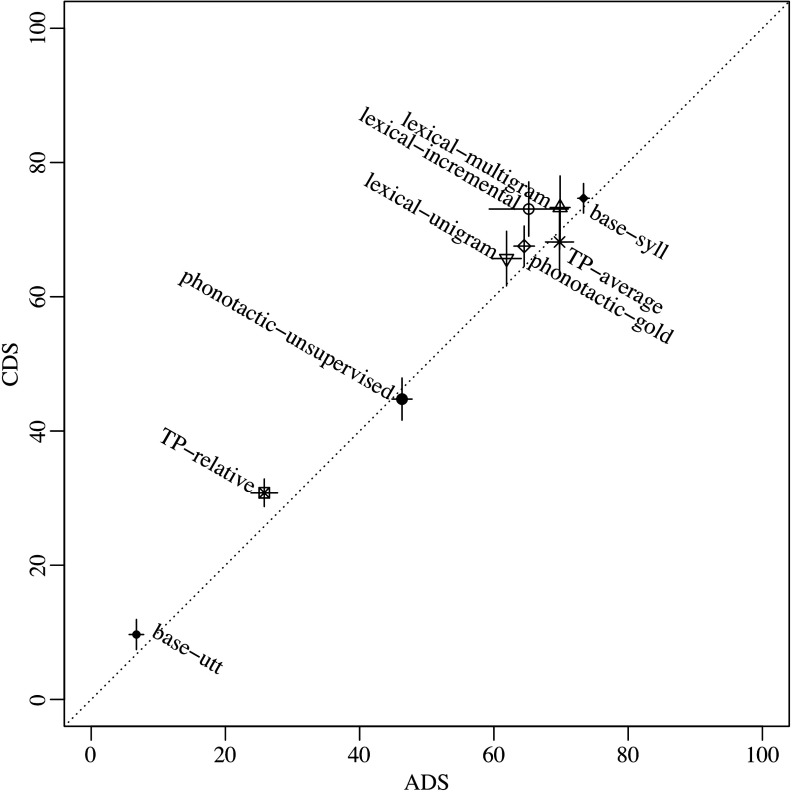
**Token F-score (in percentage) achieved by each algorithm in child-directed speech (CDS) as a function of that in adult-directed speech (ADS) in the full Winnipeg corpus with human-set utterance boundaries.** Error bars indicate two standard deviations (over 10 resamples; see main text and Supplemental Materials, Cristia, 2018a, for details).

Figure 1 illustrates token F-scores in CDS as a function of that in ADS, when using the full corpora and the human-based utterance boundaries (for figures on all other conditions and dependent variables, please see Supplemental Materials; Cristia, 2018a). If CDS input is easier to segment, then points should be above the 45-degree, equal-performance dotted line. This is the case for most points. However, the median difference across registers (CDS minus ADS, in each algorithm separately) was 3%, ranging from −2% to 8%. Moreover, for most points, the pseudo-confidence intervals (defined as two times the standard deviation over 10 samples) cross the equal performance line, meaning that only for lexical-incremental, TP-relative, and base-utt are differences above this measure of sampling error. Finally, Figure 1 conveys register differences in the larger context: The greatest source of variation in performance clearly is due to the different algorithms, with token F-scores for the full CDS corpus ranging from 10% to 75%. This 65% difference is much greater than the CDS–ADS differences (maximally 8%).

How stable are these differences as a function of utterance-boundary and size-matching decisions? We looked at performance in various conditions, varying whether utterance boundaries were purely automatic (which is less likely to reflect human-coder bias than human-utterance boundary placement) and whether CDS and ADS were matched in length (since several algorithms’ performance is affected by corpus size). Positive difference scores, indicative of better CDS than ADS performance, were found in most matching conditions, regardless of whether automatic or human-utterance boundaries were used (Table 2). However, phonotactic-unsupervised and TP-average showed a consistent CDS disadvantage in all boundary and matching conditions. Moreover, the difference between CDS and ADS was reduced when considering automatic rather than human-utterance boundaries; and length-matched CDS rather than the full CDS.

**Table 2.** CDS F-score minus ADS F-score (in percentages) by algorithm, type of match, and whether human (H) or automatic (A) utterance boundaries were considered.

**Algo**	**H: full**	**H: UM**	**H: WM**	**A: full**	**A: UM**	**A: WM**	**Median**
base-utt	2.9	1.4	1.5	3.3	1.8	1.9	1.85
base-syll	1.3	−0.2	0	1.2	−0.2	−0.3	−0.1
phonotactic-unsupervised	−1.6	−2.5	−2.5	−1.3	−2.3	−2.4	−2.35
phonotactic-gold	3	2.6	2.8	3.1	2.8	2.8	2.8
TP-relative	5	0.7	0.9	5	0.4	0.7	0.8
TP-average	−1.6	−2.9	−2.8	−1.5	−2.9	−3.1	−2.85
lexical-incremental	7.9	−0.6	1.6	7.1	1.2	2.3	1.95
lexical-unigram	3.8	2.9	3.6	2.8	2.3	2.4	2.85
lexical-multigram	3.4	1.9	0.2	3.1	−0.8	0.4	1.15
Median	3	0.7	0.9	3.1	0.4	0.7	1.15

*Note*. Full means the full child-directed speech (CDS) corpus was used; UM = utterance match: CDS corpus shortened to have as many utterances as the adult-directed speech (ADS) corpus; WM = word match: idem for words.

In short, we observe smaller CDS advantages than those found in previous work. To check whether this was due to algorithms or corpora, we applied our extensive suite of algorithms onto the Bernstein Ratner corpus (analyzed by Batchelder, 2002). The results showed a more consistent and larger CDS advantage than in the Winnipeg corpus (median of 6%, range −2–17%; see details in the Supplemental Materials; Cristia, 2018a).

## DISCUSSION

Previous computational work using laboratory-based CDS and ADS corpora have documented an impressive CDS advantage in segmentability (15% in Batchelder, 2002—although reduced to 6% when more varied algorithms are considered; 10% in Ludusan et al., 2017). However, when applying these diverse segmentation algorithms to an ecological CDS–ADS corpus, the evidence of increased segmentability for CDS than ADS was less compelling. The CDS advantage was numerically small (median of 3%), and often within the margin of error estimated via resampling (1–6%). These conclusions were based on the full CDS and ADS corpora, with human-coded utterance boundaries, where the CDS performance was based on twice the input and potentially biased utterance-boundary decisions. The CDS advantage was even smaller when considering length-matched corpora with automatic utterance boundaries (medians of 0.4–0.7%).

A key strength of the present work lies in the use of a unique corpus, in which both CDS and ADS were collected from the children’s everyday input. It is unlikely that the difference in conclusions drawn by previous authors and those we draw is due to corpus size or child age (see Table 3; note also that we and previous authors all considered corpus size differences in some analyses). Instead, the most salient difference is the setting of the recording, which in our case is at home or in the daycare, and the fact that our ADS arises naturally in this context, rather than in an interview-like situation with an experimenter. By sampling from the home and two types of daycare environments, the CDS is likely to represent a wide range of interactions between children and a variety of caregivers, and the ADS captures speech between colleagues (e.g., professional carers in the daycares), partners (e.g., mother and father in the home), and other adult relationships (e.g., visitor, delivery person, interlocutor over the phone). Note that our ADS is only representative of the ADS present in infants’ input rather than all ADS styles (from presidential speech to intimate bedside conversations). Another interesting feature of the Winnipeg corpus is that its automatic annotation contains utterance boundaries defined bottom-up (using talker switch or lengthy pauses). These features lead us to argue that our results represent the input naturally available to English-learning Canadian children well, and, in this input, CDS and ADS do not differ greatly in word segmentation learnability. Our results are compatible with the hypothesis proposed by Benders (2013), among others, whereby CDS is shaped less by the caregivers’ attempt to specifically promote language acquisition than other potential functions (such as communicating affect).

**Table 3.** Characteristics of ADS and CDS studied in past and present work.

**Corpus**	**Addressee(s)**	**Tokens**	**Types**	**Utterances**
Bernstein Ratner	Experimenter	19,753	1,797	2,668
	Children 9–27 months	30,996	1,501	8,252
Riken	Experimenter	22,844	2,022	3,582
	Children 18–24 months	51,315	2,850	14,570
Winnipeg	Adults	8,224	1,342	1,772
	Children 13–38 months	20,786	2,015	5,320

*Note*. ADS = adult-directed speech; CDS = child-directed speech; MTTR for the Bernstein Ratner ADS was .93; CDS .88.

Another strength of this work is that we employed multiple word segmentation algorithms. This is important not only because results change even as minor parameters are set but also because there is no clear evidence as to which algorithm infants use. Children may even take advantage of diverse procedures depending on context and previous experience, for example, using transition probabilities when nothing else is available (Saffran et al., 1996) and utilizing their budding lexicon when probabilities are less clear (Mersad & Nazzi, 2012). Increasing the diversity of algorithms allows us to revise Ludusan and colleagues’ (2017) conclusion that there may be greater CDS advantages when using local cues (which perform overall worse, at about 30% Token F-score in the Riken corpus) rather than lexical algorithms (with performance at about 50%). In contrast, we find that sublexical algorithms can lead to poor or good performances depending on their parametrization (compare phonotactic-gold versus phonotactic-unsupervised; TP-average versus TP-relative; base-utt versus base-syll). Further, we do not see larger CDS advantages for better performing or lexical algorithms compared to worse performing or sublexical algorithms. In fact, we see divergences even within two versions of the same algorithm, with, for example, phonotactic-gold and TP-relative leading to a CDS advantage, whereas phonotactic-unsupervised and TP-average lead to a CDS disadvantage.

We see two promising paths that future computational work should take. First, even though our algorithms covered a wide range of hypotheses regarding early word segmentation, they may differ in critical ways from the algorithms and input used by infants. For example, words here were systematically attributed a pronunciation from a dictionary, and thus did not capture the possible application of phonological rules and other sources of variation that cause a single underlying word to have many different surface forms (see Buckler, Goy, & Johnson, 2018, for phonetic variability in CDS versus ADS differently; and Elsner, Goldwater, Feldman, & Wood, 2013, for a possible incorporation of phonetic variability in segmentation algorithms). Such variability will most greatly affect the discovery of paradigms (i.e., figuring out that “what is that” can also be pronounced “whaz that”), and not necessarily segmentation of word forms. Therefore, it would be most interesting to study it in the context of morphological discovery rather than only segmentation. Ultimately, we may want to test algorithms that operate directly from the acoustic representation (Ludusan et al., 2014; Versteegh et al., 2015).

Second, we studied only North American English. We look forward to extending the current approach to ecologically valid databases in additional typologically diverse languages, although none containing both CDS and ADS is currently available, and therefore a priority in future research should be to build larger, matched, multilingual corpora. We predict segmentation scores are lower in languages where words and syllable boundaries are less well-aligned than in English (Loukatou, Stoll, Blasi, & Cristia, 2018), but regardless of overall performance levels, there will be no or little learnability advantages for CDS versus ADS for segmentation: North American English has been described as having more marked CDS–ADS differences than other languages (e.g., Japanese; Fernald et al., 1989). Therefore, one might expect the greatest learnability advantages to be found in North American English—suggesting that cross-linguistic work is even less likely to find results supporting a segmentation advantage for CDS.

To conclude, we found that advantages in segmentability for CDS over ADS in an ecological corpus were smaller and more inconsistent than previous estimations based on laboratory CDS–ADS. Overall, our word segmentation results align with other work on sound discriminability (Martin et al., 2015) and word discriminability (Guevara-Rukoz et al., 2018), suggesting that the high learnability attributed to CDS may have been overestimated. Research assessing the learnability properties of child-directed speech at other levels (e.g., syntax) would benefit from using similarly natural corpora, as well as a variety of algorithmic approaches.

## ACKNOWLEDGMENTS

We are grateful to Mark Johnson, Robert Daland, and Amanda Saksida for helpful discussions and comments on previous versions of this manuscript; and to members of the LAAC, CoML, and Language teams at the LSCP for helpful discussion.

## FUNDING INFORMATION

AC acknowledges financial support from Agence Nationale de la Recherche (ANR-14-CE30-0003 MechELex); ED from European Research Council (ERC-2011-AdG-295810 BOOTPHON), the Fondation de France, the Ecole de Neurosciences de Paris, the Region Ile de France (DIM cerveau et pensée); MS from SSHRC (Insight Development Grant 430-2011-0459, and Insight Grant 435-2015-0628). AC and ED acknowledge the institutional support of Agence Nationale de la Recherche (ANR-17-EURE-0017).

## AUTHOR CONTRIBUTIONS

AC: Conceptualization: Lead; Data curation: Lead; Formal analysis: Lead; Funding acquisition: Lead; Methodology: Lead; Project administration: Lead; Resources: Lead; Software: Lead; Validation: Lead; Visualization: Lead; Writing – original draft: Lead; Writing – review & editing: Lead. ED: Conceptualization: Supporting; Formal analysis: Supporting; Methodology: Supporting; Software: Supporting; Visualization: Supporting; Writing – review & editing: Supporting. NBR: Conceptualization: Supporting; Resources: Supporting; Writing – review & editing: Supporting. MS: Conceptualization: Supporting; Methodology: Supporting; Resources: Lead; Validation: Supporting; Visualization: Supporting; Writing – review & editing: Supporting.

## Notes

^1^ The LENA Foundation built a hardware and software system to record and automatically analyze day-long child-centered recordings. For more information, see Soderstrom and Wittebolle (2013).^2^ The transcription manual is available from https://osf.io/rvdbq/.
